# Open Science and Reporting Animal Studies: Who's Accountable?

**DOI:** 10.1371/journal.pbio.1001757

**Published:** 2014-01-07

**Authors:** Jonathan A. Eisen, Emma Ganley, Catriona J. MacCallum

**Affiliations:** 1 University of California Davis, Davis, California, United States of America; 2 Public Library of Science, Cambridge, United Kingdom

## Abstract

If being open means maximizing the number of people a paper can reach and minimizing the difficulties of re-using the information within it, then the release of all information associated with a paper is critical. For ethical reasons, high standards of reporting are extra critical in regards to animal research.

What responsibility do scientists have to report the experimental work and analyses they do on animals fully and transparently, and what responsibilities do funders, journal editors, and reviewers have to ensure that what is reported is done so appropriately? While the answer to both of these questions might seem obvious, the accumulating evidence suggests that the actual reporting done in publications is far from ideal. As Baker et al. discuss in this issue of *PLOS Biology* [1], 86%–87% of experimental articles do not give any indication that the animals in the study were properly randomized, and 95% do not demonstrate that their study had a sample size sufficient to detect an effect of the treatment were there to be one [2],[3]. Moreover, they show that 13% of studies of rodents with experimental autoimmune encephalomyelitis (an animal model of multiple sclerosis) failed to report any statistical analyses at all, and 55% included inappropriate statistics [1]. And while you might expect that publications in “higher ranked” journals would have better reporting and a more rigorous methodology, Baker et al. reveal that higher ranked journals (with an impact factor greater than ten) are twice as likely to report either no or inappropriate statistics.

Poor reporting has at least four equally serious, interconnected consequences (e.g., [2]–[11]). The first is increasing evidence that experimental pre-clinical work purportedly demonstrating the impact of a particular drug or intervention on an animal model fails in translation [12],[13]: follow-up clinical work in humans shows either no effect, for example, or that there are side effects that were never detected in the animal model. The second is that “underpowered” studies—designed with too small a sample size—will either be too small to detect an effect, which may result in a false-negative result, or will demonstrate effects only by chance—a false positive. Underpowered studies can be cumulatively useful for inclusion in meta-analyses of interventions, but only if they are reported fully and properly. The third consequence of poor reporting is that badly reported studies cannot be validated—there are often insufficient details to replicate the study design or analyses. Finally, poor reporting leads to publication bias: the pressure to publish only positive results means that negative studies are not reported or there is a bias to include selective analyses that report significant effects [3],[6],[8].

For research, if being “open” means maximizing the number of people an article can reach and minimizing the difficulties that readers have in using the information within it [14], then full and transparent reporting is a fundamental component of open access and open science. The ability to build on any study—to reuse information from any of its component parts—relies on accurate reporting of all the methods and analyses, and full release of any and all data and results from the study. While we can most easily see the consequences for pre-clinical experimental work on animals, the principle applies to any type of study or hypothesis tested. Where animals are involved, however, there is also an ethical obligation to ensure that the work is sound and the animals are not wasted (even if that counterintuitively means increasing the sample size of animals used).

PLOS (and other publishers such as Nature research journals and BioMed Central) endorsed the use of a set of standardized reporting guidelines for animal studies involving in vivo experiments—the ARRIVE guidelines [15]—in 2010. As noted in an editorial in *PLOS Biology* at the time [16], while we encouraged authors of articles published in PLOS journals to follow them, we did not mandate their use. In this issue, Baker et al. show that the guidelines had little impact on how well experimental details were reported in experimental autoimmune encephalomyelitis studies in PLOS journals (primarily *PLOS ONE*) and *Nature* in the two years after introducing the guidelines, compared with the previous two years.

The authors note that endorsing the guidelines is meaningless unless journals actually implement them. They also conclude that asking authors and journals to implement all 20 items of the ARRIVE guidelines is outside the current reporting norms in biology and that some of the guidelines are already incorporated as part of the standard institutional ethical review process and need not be additionally reported in published articles. Based on analyses of neurological studies, Baker et al. [1],[17] and Landis et al. [13] have therefore called for a core set of reporting standards that includes randomization, blinding, sample-size estimation, and the handling of all data. Some journals, such as Nature research journals, have recently mandated a customized checklist that includes some of these elements in response [18].

PLOS publishes a huge number of whole-animal-related research articles, including observational as well as in vivo experimental work. The animals range from worms, beetles, and flies to horses and primates. There are hundreds of articles in *PLOS Biology* and several thousand articles in *PLOS ONE* that present in vivo experimental work. We agree with Baker et al. that to be effective, journals need to implement, rather than just endorse, reporting standards. But we also need to ensure that reporting standards are consistent within and across fields. The ARRIVE guidelines have been endorsed by 300 journals [19], different funding agencies in the UK [20], and the National Research Council Institute for Laboratory Animal Research in the US [21], and they provide a way to set community standards and help shift the consensus about reporting norms. In the UK, the Wellcome Trust, the Biotechnology and Biological Sciences Research Council, and the Medical Research Council have mandated compliance with the guidelines as a condition of funding [20]. Applying such standards has been most effective in the past when journals universally adopt them, such as international standards for describing new species (e.g., for plants [22]) or the CONSORT guidelines for reporting randomized controlled clinical trials (http://www.consort-statement.org/). If the guidelines are held and maintained by an independent and publicly funded body (such as the National Centre for the Replacement, Refinement and Reduction of Animals in Research (http://www.nc3rs.org.uk/), which hosts the ARRIVE guidelines), they are easier to update, easier for authors or reviewers to find, and easier for authors and funders to refer to in a variety of research outputs (e.g., in grant applications as well as articles). PLOS has a dedicated collection that highlights articles that have discussed guidelines or include related research on guidelines [23], and *PLOS ONE* recently discussed on its blog how staff check that articles are reported appropriately before they are assessed by editors and reviewers [24].

Because reporting guidelines are not an accepted norm in biology, Baker et al. [1] suggest that core elements of quality experimental design and reporting be adopted in the first instance. Authors, however, should already have to hand all the information requested in the ARRIVE guidelines as an intrinsic component of their research, and there seems little justification not to include all such information as part of a publication. The absence of blinding or randomization or a power calculation in an experimental study can indicate poor experimental design [13], but it does not necessarily mean the study is wrong or should not be published or that the data cannot be used. What is missing or not missing, however, must be reported so that others can make use of the information appropriately. Likewise, there is no reason not to include standard statements about the housing or welfare or gender of the animals involved—gender-specific outcomes are well known, but other effects are less well known: in some studies differences in housing affected the way animals responded to analgesics [4],[25]. Unless such variables are listed, there will be no opportunity to test such differences or include the study in a meta-analysis, where the conditions need to be the same. In general, release of all information associated with an article is critical for any type of research, but it seems to us that, for ethical reasons, it is extra critical in regards to animal research. These are not difficult or unreasonable requests. Authors, funders, and journals are all accountable in this process—supporting good science and open access has never been simpler.

The ARRIVE guidelines now come with a useful checklist for authors [26], which authors can submit alongside their manuscript with the relevant sections completed. All the journals at PLOS are editorially independent and are making their own decisions about whether and how to implement the guidelines. *PLOS Medicine* has already mandated that authors submit the checklist for the few relevant articles it publishes [27], and *PLOS Medicine* editors will take the checklist items into account as they evaluate the research. *PLOS ONE* is currently encouraging the use of the checklist and is likely to mandate its inclusion in all submissions reporting experimental in vivo work starting in 2014. The checklist will be made available to editors and reviewers. At *PLOS Biology*, we are discussing how best to proceed with members of our Editorial Board and will work with our authors to include correct and comprehensive reporting of all the necessary experimental information. We strongly encourage authors to submit the completed checklist to enhance reusability of data, and we welcome feedback on this from the community.
